# Neuroplastic effects of transcranial near-infrared stimulation (tNIRS) on the motor cortex

**DOI:** 10.3389/fnbeh.2015.00147

**Published:** 2015-06-02

**Authors:** Leila Chaieb, Andrea Antal, Florentin Masurat, Walter Paulus

**Affiliations:** ^1^Department of Clinical Neurophysiology, Georg-August UniversityGöttingen, Germany; ^2^Department of Epileptology, University of BonnBonn, Germany

**Keywords:** neuroplasticity, transcranial near-infrared laser light stimulation, brain, learning, human

## Abstract

Near-infrared light stimulation of the brain has been claimed to improve deficits caused by traumatic brain injury and stroke. Here, we exploit the effect of transcranial near-infrared stimulation (tNIRS) as a tool to modulate cortical excitability in the healthy human brain. tNIRS was applied at a wavelength of 810 nm for 10 min over the hand area of the primary motor cortex (M1). Both single-pulse and paired-pulse measures of transcranial magnetic stimulation (TMS) were used to assess levels of cortical excitability in the corticospinal pathway and intracortical circuits. The serial reaction time task (SRTT) was used to investigate the possible effect of tNIRS on implicit learning. By evaluating the mean amplitude of single-pulse TMS elicited motor-evoked-potentials (MEPs) a significant decrease of the amplitude was observed up to 30 min post-stimulation, compared to baseline. Furthermore, the short interval cortical inhibition (SICI) was increased and facilitation (ICF) decreased significantly after tNIRS. The results from the SRTT experiment show that there was no net effect of stimulation on the performance of the participants. Results of a study questionnaire demonstrated that tNIRS did not induce serious side effects apart from light headache and fatigue. Nevertheless, 66% were able to detect the difference between active and sham stimulation conditions. In this study we provide further evidence that tNIRS is suitable as a tool for influencing cortical excitability and activity in the healthy human brain.

## Introduction

The transcranial application of near infrared light (tNIRS) to tissues in both the peripheral and the central nervous system (CNS) has been performed for at least a decade and stimulation parameters like wavelength, fluence, irradiance, treatment duration and timing, continuous or pulsed stream of laser light have been investigated, mainly in animal models (Bjordal et al., 2003; Ilic et al., 2006; Huang et al., 2009). A U-shaped response curve characterizes the optimum dosage of laser light promoting wound healing and regeneration of tissue, while a higher dosage has a detrimental effect due to heating of the tissue (Huang et al., 2009; Hashmi et al., 2010a,b; Chung et al., 2012). The primary target of applying infrared light as a therapeutic tool is for wound healing, inflammation and chronic pain relief. Applications have been widened to include the potential of rehabilitative treatment for neurological disorders, which has been extensively investigated using animal models (Detaboada et al., 2006; Oron et al., 2006, 2012), in clinical trials of patients with stroke and traumatic brain injury (Lampl et al., 2007; Hashmi et al., 2010a; Stemer et al., 2010; Naeser et al., 2011; Zivin et al., 2014), as well as showing promise as a potential treatment for Alzheimer's disease (Sommer et al., 2012). tNIRS therapies, applied in optimized dosages have been claimed to produce remarkable and reproducible effects both in the brain and peripheral tissues after traumatic insult in both animal models of disease and in humans (Gigo-Benato et al., 2005; Ilic et al., 2006; Naeser et al., 2011). The outcomes of these studies have led to the establishment of a multinational stroke trial (NCT01120301) to investigate the application of tNIRS in stroke rehabilitation and its ability to limit cognitive deficits post stroke onset (Lampl et al., 2007; Stemer et al., 2010; Zivin et al., 2014).

The putative mechanism of action of infrared light is believed potentiate the cytochrome C oxidase (CCO or complex IV) complex in the mitochondria, a component of the electron transport chain and key complex in ATP production. The action spectrum of CCO is in the near-infrared range. As tNIRS is applied at a wavelength of 810 nm, this suggests that CCO might play a key role in the cellular response of the stimulation (Karu, 1987). *In vitro* experiments have shown that laser irradiation modulates mitochondrial respiration levels, and is increased following irradiation of cellular tissues, causing an amplification of mitochondrial products, such as ATP, nicotinamide adenine dinucleotide (NADH), protein and ribonucleic acid (RNA) (Karu, 1999). tNIRS could increase the process of cellular respiration in neurons by increasing energy and cyclic adenosine monophosphate (cAMP) levels and indirectly, modulate the activity of neurons. Konstantinovic et al. (2013) in a previous study extended this view by highlighting the role of changing intracellular calcium concentration due to cortical trauma, and the modulation of Na^+^K^+^—ATPase activity associated with neurological pathologies, like stroke and traumatic brain injury. They hypothesized that application of tNIRS has a membrane stabilizing effect and (the increased activity of the Na^+^ pumps due to laser light irradiation underlies these stabilization effects) that may be an important contributing factor behind the positive clinical effects reported in earlier acute stroke studies. Next to the potential role of CCO in the effect of tNIRS a second putative mechanism of how near-infrared light can affect neurons is through the dissociation of nitric oxide (NO) and oxygen (Hashmi et al., 2010a). NO is an important cellular signaling molecule, and is also a potent neurotransmitter in the CNS, which is capable of inducing synaptic plasticity (Iino, 2006). By the action of laser induced NO dissociation from the CCO complex, the ongoing cellular respiration rate in the mitochondria can continue unhindered, even under conditions of stress (Karu, 1989).

tNIRS is technically similar to the near-infrared spectroscopy (NIRS) that is a widely applied non-invasive method for studying functional activation through monitoring changes in the hemodynamic properties of the brain, at least with regard to the wavelength of the applied light (Villringer et al., 1993). However, in the case of NIRS the power level of the stimulation is highly depends on the type of application and the number sources (up to 500 mW).

Here, we provide evidence that tNIRS is suitable as a tool for influencing cortical excitability and activity in the healthy human brain. A previous study has already reported that infrared stimulation can decrease motor cortical excitability in healthy subjects (Konstantinovic et al., 2013). In order to replicate and extend these data we have applied tNIRS over the cortical representation of the hand area of the primary motor cortex (M1) using a constellation of four laser diodes attached to percutaneous acupuncture needles. With this study we aimed to investigate whether tNIRS was (i) able to modulate patterns of cortical excitability (single-pulse measures of cortical excitability); (ii) which intracortical neural circuits were affected by this form of stimulation using paired-pulse measures; and lastly (iii) whether any change in performance on the behavioral or cognitive levels could be detected (using the SRTT). This final objective is very relevant to studies investigating the effects of near-infrared laser light stimulation on the intact and damaged cortex in patients suffering from stroke related pathologies or patients who have been treated for traumatic brain injury (Gur et al., 2007; Lampl et al., 2007; Hashmi et al., 2010a; Naeser et al., 2011).

## Materials and methods

The study was approved by the ethics committee of the University of Göttingen and conformed to the Declaration of Helsinki. All participants were informed as to all aspects of the experiments and gave written consent.

### Subjects

Altogether 55 right handed volunteers in the age range of 18–35 years were recruited, passed a standard physician's examination and met further inclusion criteria: no neurological or psychiatric disorders, pacemaker, metal implants in the head region, pregnancy, drug or alcohol addiction, or participation in another study within the last 6 weeks.

### Transcranial near-infrared laser stimulation (tNIRS)

tNIRS was applied using a continuous wave diverging laser beam, with an increase in diameter of the beam width of 2 mm with every 1 cm increase along its path length. There are currently no protocols exist in a healthy population, in which the factors (intensity, power, duration, and fluence of the laser light etc.) of the stimulation are defined in detail. Therefore, there is no consensus as to which parameters should be selected for stimulation of the intact cortex in order for the near-infrared laser light to optimally stimulate the target cortical area. According to our laboratory measurements and data from a previous study (Litscher and Litscher, 2013), the penetration of the infrared light through the skull (6–7 mm thickness) is about 1–5%. Depending on the thickness of the skin (for which every mm half of the irradiated energy of the beam is absorbed) and cerebrospinal fluid (CSF), only a small fraction of the emitted laser light energy can reach the cortical surface. Our stimulation parameters were thus: we have used a total power of 150 mW over an area of 0.35 cm^2^, which equates to a power density of 500 mW/cm^2^ on the surface of the skin, resulting in less than 5 mW/cm^2^ cortical fluence (~ 1 J total energy). The temperature increase on the skin under the diodes was max 1°C. This value is lower than can be measured during the application of other NIR light-based applications, such as pulse oximetry, NIRS and diffuse optical tomography (Bozkurt and Onaral, 2004). In previous studies similar stimulation intensity values were used with an even longer stimulation duration (20 min) applying stimulation over the center of the scalp for treating burnout syndrome (Litscher et al., 2013). Other studies investigating the treatment of patients with traumatic brain injury or depression used a 500 mW total energy dose (Naeser et al., 2011; Naeser and Hamblin, 2011) or a calculated cortical power density of 9.5 mW/cm^2^ (Schiffer et al., 2009). According to the later study the output of the device they used was “at least 5 times less than the PhotoThera laser device (personal communication, Luis DeTaboada, PhotoThera Inc, Carlsbad, CA) that was used without observed side-effects in stroke patients” (Lampl et al., 2007).

**Figure 1:**
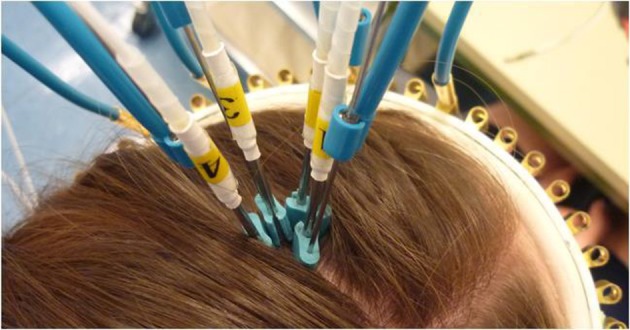
**tNIRS head montage**. The laser acupuncture needles are fixed to scalp with the crown and the bendable wire holding mechanism. The waves are carried via optical fibers to the stainless steel percutaneous needles.

tNIRS was applied using four stainless steel laser acupuncture diode needles, which were sterilized after each use. The laser needles were placed in a square over the M1, at the “hotspot” predetermined by TMS (see below) and held in place with wire holders attached to a crown that wraps around the head of the participant (Figure 1). The diodes did not touch the skin or each other, there was 5 mm distance between the skin and between the diodes. In order to exclude the unspecific effects of the stimulation, eight subjects participated in a control condition, in which the same laser needles were placed over the Oz electrode position (see below).

The laser stimulator (WeberMedical, GmbH; Klasse 1, Type BF, Laser Class 3B, max power for infrared 100 mW/diode; with certifications for human applications, including stimulation of the scalp, in the EU and USA) was programmed to administer tNIRS for 10 min; once the preset duration has been reached, the stimulation is terminated automatically. All four needles were active during stimulation producing about 150 mW energy. In each experiment subjects had to participate in 2 experimental sessions, receiving either placebo or active stimulation in a randomized counterbalanced order. During the placebo condition the laser was switched on for a 30 s period only. A minimum of 4 days were maintained between each experimental session to avoid any carry-over effects of the stimulation.

### Measurement of motor-cortical excitability

To detect changes in excitability motor-evoked-potentials (MEPs) of the right first dorsal interosseous (FDI) were recorded following tNIRS of its motor-cortical representational field by single-pulse TMS. These were elicited using a Magstim 200 magnetic stimulator (Magstim, Whiteland, Dyfed, UK) and a figure-of-eight magnetic coil (diameter of one winding = 70 mm; peak magnetic field = 2.2 Tesla). The coil was held tangentially to the skull, with the handle pointing backwards and laterally at 45° from the midline. The optimal position was defined as the site where stimulation resulted consistently in the largest MEP. Surface electromyogram (EMG) was recorded from the right FDI with Ag–AgCl electrodes in a belly tendon montage. Raw signals were amplified, band-pass filtered (2 Hz–3 kHz; sampling rate 5 kHz), digitized with a micro 1401 AD converter (Cambridge Electronic Design, Cambridge, UK) controlled by Signal Software (Cambridge Electronic Design, version 2.13) and stored on a personal computer for offline analysis. The intensity of the stimulator output was adjusted for baseline recording so that the average stimulus led to an MEP of 1 mV in amplitude.

Resting motor threshold (RMT), active motor threshold (AMT), the intensity required to elicit an MEP of ~1 mV peak-to-peak amplitude (SI1 mV) and a baseline of TMS-evoked MEPs at the defined SI1 mV intensity, were recorded at 0.25 Hz prior to stimulation. Stimulus intensities (in percentage of maximal stimulator output) of TMS were determined at the beginning of each experiment. RMT was defined as the minimal output of the stimulator that induced a reliable MEP (50 μV) in at least three of six consecutive trials when the FDI muscle was completely relaxed. AMT was defined as the lowest stimulus intensity at which three of six consecutive stimuli elicited reliable MEPs (200 μV) in the tonically contracting FDI muscle (Rothwell, 1999).

### Experimental procedures

We had three experimental sessions: (1) single pulse MEP measurements were introduced in order to measure corticospinal excitability (15 subjects, 7 males stimulating the M1 and 8 subjects, 3 males stimulating the visual cortex); (2) paired- pulse TMS was applied in order to measure intracortical (15 subjects, 7 males); (3) implicit motor learning task was used to test if tNIRS can modulate motor learning (32 subjects, 16 males). The experiments were conducted in a randomized, repeated measurement design, on different experimental days, separated at least with a weak pause.

#### Measuring corticospinal excitability

Before tNIRS TMS-evoked MEPs (30 stimuli) were recorded at 0.25 Hz. Baseline measurement was followed by 10 min active or sham tNIRS. After termination of tNIRS, 30 MEPs were recorded at 0 min, 5–30 min and then every 10–60 min poststimulation.

#### Measures of intracortical excitability

Short intracortical inhibition (SICI), intracortical facilitation (ICF) and long intracortical inhibition (LICI) were measured prior to active and sham stimulation sessions, immediately and 30 min poststimulation. The following protocols were used: for SICI/ICF, two magnetic stimuli were given through the same stimulating coil, and the effect of the first (conditioning) stimulus on the second (test) stimulus was investigated (Kujirai et al., 1993). To avoid any floor or ceiling effect, the intensity of the conditioning stimulus was set to 80% of AMT. The test-stimulus intensity was adjusted to SI1 mV. SICI was measured with interstimulus intervals (ISI) of 2 and 4 ms and ICF with ISIs of 7, 9, and 12 ms. At each time points the conditioning-test stimuli were recoded 20 times. The mean peak-to-peak amplitude of the conditioned MEP at each ISI was expressed as a percentage of the mean peak-to-peak size of the unconditioned test pulse.

The second protocol tested was LICI, which applies two suprathreshold stimuli with ISIs of 50, 100, 150, and 200 ms (Valls-Sole et al., 1992). The intensity of both stimuli was set to 110% of RMT. LICI was taken as the mean percentage inhibition of the conditioned test pulse MEP at ISIs of 50, 100, 150, and 200 ms. At each time points the conditioning-test stimuli were recoded 20 times.

#### Investigating implicit motor learning using a serial reaction time task (SRTT)

The SRTT (Nissen and Bullmer, 1987) is an established test to investigate implicit motor learning also in the context of brain stimulation (Nitsche et al., 2003) During the task the participant has to respond to a visual cue as fast and as accurately as possible with individual finger movements in response to a four dot sequence on the computer screen. Participants are unaware that the sequences follow a pseudo-repeating pattern, but their ability to implicitly “learn” the sequence is measured over the course of the task. The task is divided into 8 blocks. Blocks 1–5 and blocks 7 and 8 have the same pattern, whereas the sequence in block 6 is different to the other sequences presented in the other blocks. The calculated difference in the participants' reaction times in block 6 compared to their performance in block 7 is considered to be a measure of implicit motor-learning. Effects of transcranial stimulation using the SRTT have been shown to be a robust measure of this kind of learning and the structure of the paradigm ensures a specific sequence learning is measured and prevents an unspecific decreased reaction time purely due to increasing task routine (Pascual-Leone et al., 1994).

The subjects were seated in front of a computer screen placed at eye level and were not informed as to the aim of the SRTT. Their right fingers were placed on the computer keyboard on the designated keys for each finger. Four bars appeared on the screen: the first from the left corresponding to the right index finger, the second the middle finger, the third the ring finger and the fourth the little finger. The SRTT was performed using windows-based software using a modified standard keyboard in which only the buttons assigned for active button presses were present. For purpose of the task, this experimental setup was adequate for examining the differences in RTs of participants before and during tNIRS. Ten minutes tNIRS or sham stimulation was given during the performance of the task. In each trial, RT was measured from the appearance of the “go” signal until the first button was pushed by the subject. For each block of trials in a given experimental condition, mean RT was calculated for each subject separately.

### Questionnaires

To examine safety aspects and to evaluate the blinding efficacy of tNIRS, participants were asked to fill out questionnaires examining the cutaneous effects of tNIRS in the SRTT task. Side effects like heating sensations, tingling, itching and pain, fatigue, nervousness and differences in concentration as well as any other noticeable sensations were documented. The questions concerned sensations during and after (2–6 min) the stimulation. 28 questionnaires were filled out correctly (15 active and 13 sham sessions).

### Data analyses

#### Single-pulse TMS

MEP amplitude means were calculated for each time point covering baseline (30 stimuli) and poststimulation time-points (30 stimuli). Baseline normalized MEPs were analyzed using repeated measurements of ANOVA (CONDITION (tNIRS vs. sham) × TIME (0, 5, 10, 15; 20, 25; 30, 40, 50, 60 min post-stimulation). Effects were considered significant if *p* < 0.05. In the case of a significant main effect or interaction, a Student's *t*-test was performed. Student's *t*-test was used to compare the MEP values between baseline and post-stimulation measurements within group. All data are given as means + SEM.

#### Paired-pulse TMS

For each measurement [SICI, LICI, input-output curves (I/O)], we performed separate analyses of variance (ANOVAs) for repeated measurements by using the mean values from each subject as the dependent variable. In addition to the factor CONDITION (tNIRS vs. sham), the ANOVA model included the factor “ISI” when SICI/ICF (2, 4, 7, 9, 12) or LICI (50, 100, 150, 200) were analyzed. With regard to recruitment curves the factor “intensity” (100%, 130%, and 150% of RMT) was considered. A *p* value of <0.05 was considered significant for all statistical analyses. In the case of a significant main effect or interaction between ISI/intensity and stimulation condition, a Student's *t*-test was performed.

#### SRTT analysis

A repetitive measures ANOVA (independent variables: CONDITION and BLOCK) for reaction time (RT) and error rate (ER) was performed. As the RT difference between Block 5 and 6 is thought to represent an exclusive measure of implicit learning, Students' *t*-tests were performed to compare the respective differences between tNIRS and sham conditions. A p value of <0.05 was considered significant for all statistical analyses.

## Results

All of the subjects tolerated the stimulation; none of the experimental sessions were interrupted or terminated due to side effects of the stimulation.

RMT, AMT, SICI, ICF, LICI curve baseline values were compared between tNIRS and sham conditions using Student's *t*-test. There was no significant difference in any of the measurements (all ps > 0.3).

### Single-pulse MEPs

**Figure 2:**
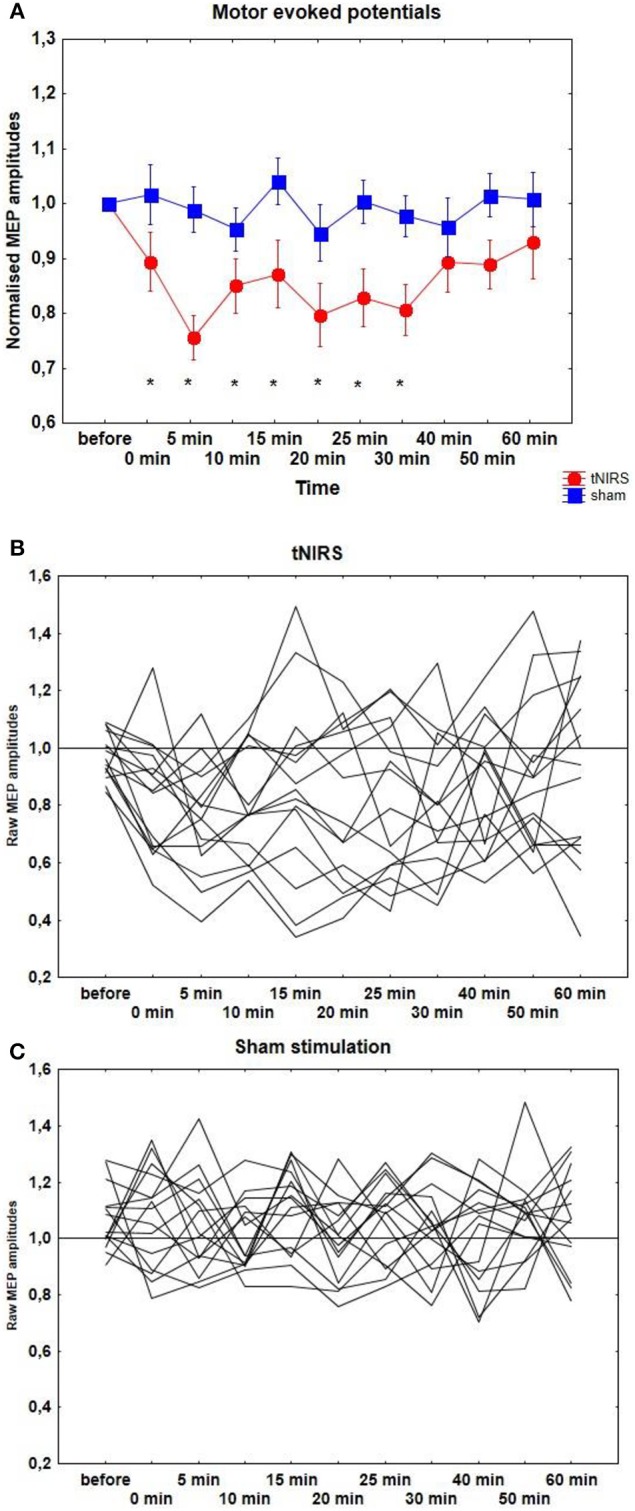
**(A)** Effect of 10 min tNIRS on motor evoked potentials. Time course of motor cortex excitability changes for 60 min post-stimulation, shown after 10 min tNIRS over M1. The figure shows mean amplitudes and their SEMs. Asterisks indicate significant differences between MEP amplitudes after 0–30 min post-stimulation compared to baseline (Student's *t*-test, *p* < 0.05). **(B,C)** Individual MEP data after active and sham stimulation.

After 10 min tNIRS cortical excitability decreased by 20–30%, as revealed by single-pulse TMS. According to the *t*-test, significantly decreased MEPs were observed at the 0 and 30 min compared to the baseline (*p* < 0.05). Repeated measurements of ANOVA revealed a significant main effect of CONDITION [*F*_(1, 14)_ = 10.21, *p* = 0.006]. The main effect of TIME [*F*_(9, 126)_ = 1.33, *p* = 0.23] and the interaction between CONDITION and TIME were not significant [*F*_(9, 126)_ = 0.73, *p* = 0.67] (Figure 2A). Individual data can be seen on Figures 2B,C.

The stimulation of the visual area did not result in any MEP change, compared to the sham condition [CONDITION: *F*_(1, 7)_ = 0.21, *p* = 0.66; TIME: *F*_(9, 63)_ = 0.73, *p* = 0.68; CONDITION × TIME: *F*_(9, 63)_ = 1.21, *p* = 0.3].

### Paired-pulse TMS

**Figure 3:**
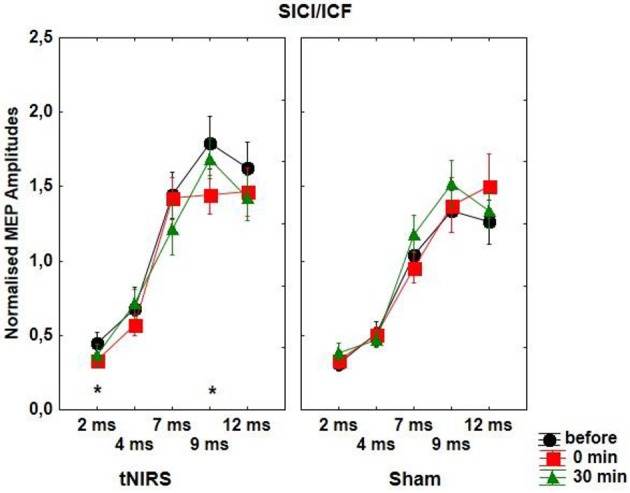
**Effect of 10 min tNIRS on SICI/ICF**. The figure shows mean amplitudes and their SEMs. Asterisks indicate significant differences between MEP amplitudes after 0 min post-stimulation compared to baseline (Student' *t*-test, ISI of 2 ms; *t* = 2.48, *p* = 0.028; ISI of 9 ms; *t* = 3.58, *p* = 0.0037).

With regard to SICI repeated measurements of ANOVA revealed a significant effect of ISI [*F*_(4, 48)_ = 63.81, *p* < 0.001] and CONDITION [*F*_(1, 12)_ = 7.99, *p* = 0.015], which was due to the significantly increased inhibition immediately at the end of the tNIRS at the ISI of 2 ms (*t* = 2.48, *p* = 0.028) and decreased excitation at the ISI of 9 ms (*t* = 3.58, *p* = 0.0037) (Figure 3). There were no other significant main or interaction effects with regard to SICF/ICF.

tNIRS had no significant effect on LICI and motor-evoked recruitment curves as revealed by repeated measurements of ANOVA.

### Implicit motor learning

Repeated measures ANOVA revealed a significant main effect on BLOCK [*F*_(7, 217)_ = 22.20, *p* < 0.001] There was no significant effect on CONDITION [*F*_(1, 31)_ = 0.2, *p* = 0.66] and the CONDITION × BLOCK interaction was also not significant [*F*_(7, 217)_ = 0.43, *p* = 0.88].

With regard to the ER, repeated measures ANOVA revealed a significant main effect on BLOCK [*F*_(7, 217)_ = 17.26 *p* < 0.001] There was no significant effect on CONDITION [*F*_(1, 31)_ = 0.13, *p* = 0.72] and the CONDITION × BLOCK interaction was also not significant [*F*_(7, 217)_ = 0.53, *p* = 0.16].

### Perceptual sensations and side effects during and after stimulation

During active stimulation 100% of the subjects reported feeling a heating sensation during active and 7.7% of them during sham stimulation (significant difference between active and sham stimulation, Chi-square test *p* < 0.001) (Table 1). Pain and tingling were reported by 60.3% and 46.9% of the subjects respectively (significant difference between active and sham stimulation, Chi-square test *p* < 0.001). Fatigue was higher in the sham group (38.5% vs. 26.8%) during stimulation. 66% of the subjects were able to distinguish between sham and active stimulation. After active stimulation 26.8% of the subjects experienced heating and pain sensations. Itching and tingling sensations were similar in both groups (between 7.7% and 15.4%). Light headache was reported by 19.8% of the participants.

**Table 1:** **Perceptual and side effects of the stimulation**.

	**Tingling**		**Itching sensation**	
	**During%**	**After%**	**During%**	**After%**
active	46.9	13.4	13.2	13.2
sham	7.7	7.7	0	15.4
	**Heating sensation**		**Pain**	
	**During%**	**After%**	**During%**	**After%**
active	100	26.8	60.3	26.8
sham	7.7	7.7	0	0
	**Headache**		**Fatigue**	
	**During%**	**After%**	**During%**	**After%**
active	6.6	19.8	26.8	39.6
sham	7.7	0	38.5	30.8
	**Change in visual perception**		**Nervousness**	
	**During%**	**After%**	**During%**	**After%**
active	6.6	6.6	19.8	0
sham	15.4	7.7	7.7	0

*N = 15 active, 13 sham conditions*.

## Discussion

Earlier works using NIRS as a measurement tool, have already demonstrated that near-infrared light can penetrate the intact skull and reaches deeper tissue than red light (Chung et al., 2012). In the present study, supporting previous findings (Konstantinovic et al., 2013), we have shown that a 10 min. application of tNIRS to the M1 can inhibit cortical excitability as measured by attenuation of the amplitude of TMS-elicited MEPs. The duration of the induced inhibition was longer than the stimulation itself: the MEP amplitudes reached baseline values after 30 min poststimulation. We have further observed to an increased SICI and a decreased ICF after active stimulation. SICI reflects intracortical inhibition and is mediated by gamma aminobutyric acid (GABA_A_) receptors, whereas ICF is most likely mediated by the glutamatergic system (Ziemann et al., 1998). Therefore, it is possible that tNIRS facilitated intracortical inhibitory networks and/or inhibited intracortical facilitatory influences of corticospinal motoneurons, by increasing GABAergic neurotransmission and/or decreasing glutamatergic actions, thus resulting in a net inhibition of MEP amplitudes. Evidence for an earlier appearance or predominance of inhibition using other transcranial stimulation methods (e.g., electrical stimulation) was already published in human (Moliadze et al., 2012) and animal studies (Le Roux et al., 2006, 2008). On the neuronal level nonlinear excitation-inhibition integration caused by shunting of excitatory synaptic currents through activated GABA_A_ channels has been shown experimentally (Borg-Graham et al., 1998; Hao et al., 2009) and theoretically (Blomfield, 1974; Koch et al., 1983; Hao et al., 2009). Moreover, it was shown that excitatory circuits are strongly controlled by inhibitory circuits (Maffei et al., 2004). On the molecular level the reactive oxygen species (ROS)-pathway might also play a possible role in this process. Increased cellular respiration and increased oxygen consumption follow rises of intracellular ROS (Storz, 2007), which in turn, increases the overall redox potential of the cell. However, considering our stimulation duration (10 min) it can be that the products of upregulated respiration (ATP) or even the mitochondria themselves begin to downregulate and that the normally functioning GABAergic mechanisms override the already dysfacilitated excitatory circuits. It would be an important question to investigate whether these effects are due to a reduction in the activity of the mitochondria in targeted neurons.

On the behavioral level using the SRTT task we have observed no significant effect of tNIRS on the implicit learning process. This is a partly contradictory result compared to the inhibitory effect of tNIRS that we observed on the MEP amplitude. However, dissociation between MEP excitability changes and implicit learning using electrical stimulation has already been described (Antal et al., 2008; Moliadze et al., 2010). In MEP measurements and in implicit motor learning different anatomical pathways and physiological processes are involved that may reflect the involvement of diverse neuronal populations.

The study has several limitations. The most important point is, that a high percentage of participants reported cutaneous perceptions, including a heating sensation during stimulation and therefore, were able to differentiate between the active and sham stimulation conditions, which in turn, might influence the present results. In an earlier study, suppression of MEPs was observed after painful infusion of hypertonic saline into the hand muscle (Svensson et al., 2003); nevertheless, here the acute pain was induced in the muscle, from which the MEPs were recorded. Generally, positive and negative emotions (like pain) (Hajcak et al., 2007) and increased attention toward the experimental procedure (Stefan et al., 2004) have been suggested to *increase* and not to decrease MEP amplitude. Furthermore, the stimulation had an aftereffect, the MEP size reached the baseline level in ca 20 min after the end of the stimulation that is very unlikely the effect of acute local tingling and heating sensations. Finally, in our control condition, where the visual cortex was stimulated in 8 subjects, the participants experienced the same skin sensations like during M1 stimulation, however, we did not observe any MEP amplitude change. Therefore, we are convinced that the results are real and the inhibitory effect of tNIRS is due to the M1 stimulation. Nevertheless, further work should be done to develop a more appropriate placebo condition. Aside from this it is of utmost priority to minimize any accompanying cutaneous sensations.

The second point is that the individual variability with regard to the cortical excitability changes (that might be the reason of the missing effect of the stimulation in the implicit learning task) is high, although a clear tendency toward the inhibition can be observed. It is well documented that the penetration depth of infrared light depends on the thickness of the scalp and skull (e.g., Li et al., 2007; Yoshitani et al., 2007; Strangman et al., 2014) that can be very different in healthy subjects, resulting in altered penetration depths.

In summary, recent human and animal studies have shown that near-infrared light applied over the cortex may have beneficial effects on stroke rehabilitation and may minimize cognitive deficits sustained during traumatic brain injury (Hashmi et al., 2010a; Stemer et al., 2010; Ando et al., 2011). Here, we claim that tNIRS offers the potential to induce neuroplastic changes in the intact human cortex. Since tNIRS is believed to modify mitochondrial respiration, it might offer a possibility to aid in the management of a wide variety of disease pathologies originating from mitochondrial dysfunction.

### Conflict of interest statement

The study was partially supported by WeberMedical, GmbH. The authors declare that the research was conducted in the absence of any commercial or financial relationships that could be construed as a potential conflict of interest.

## Abbreviations

AMT: active motor thresholds
CCO: cytochrome C oxidase
EMG: electromyogram
FDI: first dorsal interosseous muscle
ICF: intracortical facilitation
ISI: interstimulus intervals
M1: primary motor cortex
MEP: motor evoked potential
RMT: resting motor threshold
SICI: short intracortical inhibition
SRTT: serial reaction time task
tACS: transcranial alternating current stimulation
tDCS: transcranial direct current stimulation
tNIRS: transcranial near-infrared stimulation
TMS: transcranial magnetic stimulation
tRNS: transcranial random noise stimulation.
